# The Disposal of Sewage of Public Edifices

**Published:** 1891-01

**Authors:** 


**The Disposal of the Sewage of Public Edifices. Circular No. 20, State Board of Health of Penna.**

This tract deals with the question of disposal of sewage from jails, almshouses, etc., which, according to present practice, is allowed to pollute streams of water. The tract is an eloquent protest against this dreadful practice. It concludes with a list of the best works to be had upon the question.

